# Barriers and Facilitating Factors for Conducting Systematic Evidence Assessments in Academic Clinical Trials

**DOI:** 10.1001/jamanetworkopen.2021.36577

**Published:** 2021-11-30

**Authors:** Stuart McLennan, Barbara Nussbaumer-Streit, Lars G. Hemkens, Matthias Briel

**Affiliations:** 1 Department of Clinical Research, Basel Institute for Clinical Epidemiology and Biostatistics, University of Basel and University Hospital Basel, Basel, Switzerland; 2 Institute of History and Ethics in Medicine, TUM School of Medicine, Technical University of Munich, Munich, Germany; 3 Cochrane Austria, Department for Evidence-based Medicine and Evaluation, Danube University Krems, Krems, Austria; 4 Meta-Research Innovation Center at Stanford, Stanford University, Stanford, California; 5 Meta-Research Innovation Center Berlin, Berlin Institute of Health, Berlin, Germany; 6 Department of Health Research Methods, Evidence, and Impact, McMaster University, Hamilton, Ontario, Canada

## Abstract

**Importance:**

A systematic assessment of existing research should justify the conduct and inform the design of new clinical research but is often lacking. There is little research on the barriers to and factors facilitating systematic evidence assessments.

**Objective:**

To examine the practices and attitudes of Swiss stakeholders and international funders regarding conducting systematic evidence assessments in academic clinical trials.

**Design, Setting, and Participants:**

In this qualitative study, individual semistructured qualitative interviews were conducted between February and August 2020 with 48 Swiss stakeholder groups (27 primary investigators, 9 funders and sponsors, 6 clinical trial support organizations, and 6 ethics committee members) and between January and March 2021 with 9 international funders of clinical trials from North America and Europe with a reputation for requiring systematic evidence synthesis in applications for academic clinical trials.

**Main Outcomes and Measures:**

The main outcomes were practices and attitudes of Swiss stakeholders and international funders regarding conducting systematic evidence assessments in academic clinical trials. Interviews were analyzed using conventional content analysis.

**Results:**

Of the 57 participants, 40 (70.2%) were male. Participants universally acknowledged that a comprehensive understanding of the previous evidence is important but reported wide variation regarding how this should be achieved. Participants reported that the conduct of formal systematic reviews was currently not expected before most clinical trials, but most international funders reported expecting a systematic search for the existing evidence. Whereas time and resources were reported by all participants as barriers to conducting systematic reviews, the Swiss research ecosystem was reported not to be as supportive of a systematic approach compared with international settings.

**Conclusions and Relevance:**

In this qualitative study, Swiss stakeholders and international funders generally agreed that new clinical trials should be justified by a systematic evidence assessment but that barriers on individual, organizational, and political levels kept them from implementing it. More explicit requirements from funders appear to be needed to clarify the required level of comprehensiveness in summarizing existing evidence for different types of clinical trials.

## Key Points

**Question:**

What are the practices and attitudes of Swiss stakeholders and international funders regarding conducting systematic evidence assessments in academic clinical trials?

**Findings:**

In this qualitative study in which 48 Swiss clinical trial stakeholders and 9 international funders were interviewed, responses varied widely regarding how previous evidence should be summarized and assessed when planning a new clinical trial. A lack of obligation, time, competent support, and financial resources was identified as a barrier for an evidence-based approach.

**Meaning:**

There may be a need for more explicit requirements from funders and ethics committees to clarify the level of comprehensiveness needed in summarizing existing evidence for different types of clinical trials.

## Teaser

This qualitative study investigates factors that may impede or facilitate inclusion of systematic evidence assessments in academic clinical trials using interviews with Swiss stakeholders and international funders.

## Introduction

New clinical research requires a systematic assessment of the existing evidence; unnecessary or poorly informed clinical research is costly and unethical, limits the available funding for relevant and well-designed research, and diminishes the public’s trust in science.^1,2,3,4,5^ A lack of a systematic assessment of prior research has led to thousands of patients being recruited into clinical trials and inadequately treated with no treatment or inferior control treatment well after the tested intervention was known to be effective.^6,7,8^ Nonsystematic reviews of the literature also often involve bias, with previous studies being selected to justify new research based on strategic considerations and preferences of the investigators, more often citing studies that have supportive, positive, and statistically significant findings vs those that have conflicting, negative, and nonsignificant findings.^9,10,11^ However, previous studies indicate that an evidence-based approach to clinical research remains insufficiently used.^12,13,14,15,16,17^

Previous research regarding the barriers and facilitating factors for using systematic reviews to justify and inform the design of new clinical trials is currently limited (search details are provided in eAppendix 1 in the Supplement). A survey conducted among delegates of the International Clinical Trials Methodology Conference in November 2015 found that time constraints were perceived as the biggest barrier to the use of systematic evidence synthesis when designing a new trial, followed by a belief that the trial was the first in the research area, a belief that previous trials were different from the current trial, financial constraints, and the fact that funders did not require systematic evidence synthesis.^18^ However, the response rate of the survey was only 17% (106 of 638 delegates), and 95% of registered delegates were from the UK and Ireland,^18^ severely limiting the validity and applicability of the survey.

In Switzerland, the need to support high-quality academic clinical trials has been increasingly recognized; a network of clinical trial support units was initiated in 2007,^19^ and the Swiss National Science Foundation has implemented a yearly program for investigator-initiated clinical trials since 2016.^20^ However, it is currently not known to what extent an evidence-based approach in academic clinical trials in Switzerland is required by funders or implemented in practice. Furthermore, previous research suggests that some international funders currently require a systematic review when applying for financial support for a new clinical trial.^21,22,23,24^ These funders are typically members of the Ensuring Value in Research Funders’ Forum,^25^ an organization aiming to help health research funders increase the value of their research. One of the principles endorsed by all members of the forum is that “Research should only be funded if set in the context of one or more existing systematic reviews of what is already known or an otherwise robust demonstration of a research gap.”^25^ However, there is a lack of research examining what these funders require in practice and their experience implementing an evidence-based approach.

Although the lack of an evidence-based approach in clinical research is well known and there is general agreement about its importance,^12,13,14,15,16,17^ it remains unclear how to best achieve widespread and sustainable implementation. A more thorough understanding of barriers and facilitating factors from the different perspectives of all relevant stakeholders is needed. This study therefore aimed to examine the practices and attitudes of Swiss stakeholders and international funders regarding conducting systematic evidence assessments in academic clinical trials.

## Methods

For this qualitative study, the study design and data collection did not require approval of an ethics committee according to Articles 1 and 2 of the Federal Act on Research Involving Human Beings in Switzerland. Verbal informed consent was obtained from participants at the start of the interview. The study followed the Consolidated Criteria for Reporting Qualitative Research (COREQ) reporting guideline.^26^ An extended methods section is provided in eAppendix 2 in the Supplement.

### Research Team and Reflexivity

Interviews were primarily conducted by S.M., a male senior researcher in biomedical ethics. One interview was conducted by M.B., a male physician and senior scientist in clinical epidemiology. Both interviewers have long-standing experience with qualitative research in the context of clinical research and evidence-based medicine.^27,28,29,30,31,32,33,34^ The interviewers had already had contact with some of the Swiss stakeholders from previous research studies. Otherwise, no relationship existed between the interviewers and the other participants before the study, and participants received limited information about interviewers.

### Study Design

Participants were primarily selected through purposive sampling^35^ to ensure sample diversity according to predetermined factors (eg, field of expertise). Additional participants were identified using snowball sampling.^36^ Participants were contacted by email and suitable dates for an interview were found with those willing to participate. The study consisted of 57 participants from 2 different samples. In the first sample, 48 Swiss stakeholders from 4 different groups were recruited: 27 primary investigators, 9 funders and sponsors, 6 clinical trial support organizations, and 6 ethics committee members. In the second sample, 9 international funders from North America and Europe with a reputation for implementing an evidence-based approach for clinical trials were recruited.^24^ Interviews were conducted between February and August 2020 with Swiss stakeholders and between January and March 2021 with international funders. One participant provided their response in writing via email; the remaining interviews were conducted via a telephone or video call. All interviews were conducted in English. Only the participant and the researcher were present during the interview. A researcher-developed semistructured interview guide was created for each group to guide the discussion (eAppendix 3 in the Supplement). The interview guide was piloted in the first 2 interviews. Because no problems were identified, no further piloting or adaptation of the interview guides was deemed to be necessary. Interviews were audio-recorded and were a mean duration of 29 minutes (range, 12-62 minutes) with Swiss stakeholders and 30 minutes (range, 22-44 minutes) with international funders. After 57 interviews, the topic of data saturation arose and was discussed by the research team.^37^ It was concluded that no new substantive themes were being expressed by the participants. Transcriptions of the interviews were returned to all participants with an invitation for them to review and send any corrections or clarifications; 8 responses were received with minor corrections to syntax.

### Data Analysis

Using the interview transcriptions in their original language, S.M. performed conventional content analysis with the assistance of the qualitative software MAXQDA, version 11 (VERBI Software). Analysis commenced while interviews were ongoing and used an iterative approach in which initial codes common across participants as well as those unique to individuals were identified using a process of open coding and developed as the analysis progressed. B.N.-S., L.G.H., and M.B. reviewed the initial analysis to clarify and refine codes, and conversations among the investigators continued until coding differences were resolved and consensus was achieved. Findings are presented as higher- and lower-level categories in a coding frame.

## Results

### Current Practice

Of the 57 participants, 40 (70.2%) were male. Swiss participants universally acknowledged the importance of having a comprehensive understanding of the previous evidence when designing and justifying a new clinical trial. However, it was reported that most investigators in Switzerland were not currently conducting systematic reviews, and it was estimated that systematic reviews only happened in 10% to 30% of trials. Many participants disagreed that systematic reviews were always necessary before a new clinical trial and reported that the need for a systematic review depended on the type of clinical trial and available evidence. Only a few participants said a systematic review should always be done before a new clinical trial if no systematic review had been recently published on the topic by other researchers (Table 1).

**Table 1.** Swiss Stakeholder Views on Reviewing the Existing Research

Theme	Example quote (participant identifier, group)
**Current practice**	
Majority of Swiss investigator-initiated clinical trials not systematically reviewing existing evidence	“To be honest, up to now I have never done a systematic review.” (P24, primary investigator) “Well, my knowledge is, all those that are consulting with us, we usually tell them that they should do a systematic review.…But I think, in general terms, I would say 20 to 30 percent max?” (P10, clinical trial support) “Yes, I think this is the vast minority, maybe 10 percent.” (P27, ethics committee)
**Barriers to a systematic approach**	
Investigator perceptions	
View that they are experts and already know the relevant research	“My subspeciality of interest [is] very narrow and therefore I was aware due to personal contacts of ongoing research projects in [the] EU and North America. In addition, I checked the clinicaltrials.gov database to make sure that no similar trial was underway.” (P1, primary investigator) “We are very much at the cutting edge of research in the field and area of our interest. We have a lot of background information of the current state of established evidence.” (P5, primary investigator)
View that the study is innovative and a systematic review does not make sense because of the limited evidence available	“So, it’s depending on the study and on the idea. People have sometimes such a new fantastic idea, which may be very innovative and not have been already done by 10 other studies, which is actually nice. Thus, a review may not really help always.” (P18, primary investigator) “No, but basically it is, the topic does not really apply to it because there [were] no clinical studies before in this field. So, it does not really fit to the topic.” (P22, primary investigator) “It depends on the question. Sometimes there is [too little] literature available [to] do a formal review. I mean, if this is a question [that] everybody is looking at, of course then you have plenty of data; then it is a different situation and then it may be that…there are already meta-analyses available. But usually, there are specific questions [for which] the context is narrow, and I do not see or we do not see a need that we ask for a formal review.” (P27, ethics committee) “A former systematic review of the literature? No, because that’s not helpful. I need to know what’s currently going on or what people are currently doing. Things that have been already published are not going to help me in designing an innovative idea.” (P34, primary investigator)
Belief that an evidence-based approach can be achieved without a systematic review	“No. No, I do not think that you always need a systematic review to do something like that. Because, for example, when you come from basic research when you go from bench to bedside, I mean, you come from a mechanistic development; you just have to check that this is not something that is already, you know, established and known, and you do not need a systematic review for that.” (P16, primary investigator) “Yes, I think to a certain extent, especially when it is in pragmatic trials. Because I think we as clinicians, or as people who are hands-on actually in daily practice, we very quickly actually see where the evidence gaps are that are really a problem to us. So…, you know, I can easily tell you 10 trials that should have been done [a long time ago]. Because we never know. And so, I am not sure; so the research idea or the trial idea or the need assessment should actually come from daily practice now in clinical trials.” (P24, primary investigator) “Well, of course, to look at the literature and see what…I’m not so convinced about so-called systematic reviews. I’ve not been convinced that they add too much to this. I have my own prejudice about these.” (P43, primary investigator)
Practical challenges	
Time consuming and time constraints	“Most of them are at these working clinics, they do it on their weekends, they do it in the evening. They don’t have like 1 week off to work on it and look on it in detail and then rewrite it. It’s always like: Add something, add something, add something. And probably they are not that well educated on how to do that.” (P3, ethics committee) “It is all just a matter of time, I mean to find to do it, yes. And especially for clinicians.” (P22, primary investigator) “One barrier is time, because a systematic review, if you do it well….They’re often not funded, and then you do it besides other work, and it can take ages. And you might not want to delay the start of the trial, because you think it’s important or because there is currently a funding opportunity. So, you have to submit by then. And there’s not enough time.” (P40, clinical trial funder)
Insufficient financial and personnel resources	“The main barrier, I would think, are resources. That is, to do a systematic review correctly, you need the expertise and the manpower…for example, data extraction, where you have the 4-eye-principle, and then also…the evidence synthesis that requires that you have a close cooperation with people that have a sound methodological background. We are really fortunate to have a close collaboration with our clinical trials unit, so for us, I’d say there is no barrier. But for others who don’t have that access, it may be a barrier in terms of cost, availability of human resources, and time constraints.” (P5, primary investigator) “The problem of investigative-instigated trials is the resources that are available, and time and money, and so on. So I think that some take shortcuts. And I think that is the reason.” (P9, ethics committee) “Funders do not have [a] possibility to…or it is very difficult to get funding for this part….[T]hey would love to see [it] in the proposal, but you will need to do it out of your own pocket, and this is a major problem. Besides the time that it requires and the qualified personnel that you need, you need to also have the resources to do it, basically.” (P12, clinical trial support)
Lack of awareness and knowledge	“I think one problem is the lack of awareness for that….They don’t immediately think of a systematic review and then lack of expertise and lack of time and resources for an appropriate funding phase of a trial. There [are] still people who have never heard of the planning grant and stuff like that. I think a combination of resources, expertise, awareness.” (P4, primary investigator) “Maybe unawareness that it would make sense. Or it could also be, of course, time-constraint. I mean, they’re [under] pressure, all these researchers; maybe they just don’t take the time to do these reviews. But I think it’s rather that they just don’t have the idea to have a review first.” (P6, clinical trial support) “Many are probably related to the competences, which are not presented in each local research unit. So, we have this decentralized competence in the clinical trial unit, but locally, there’s no such competence, even, if we’re trying to increase the degree of competences among the researchers in this field, as we are actively promoting a course on systematic review, which is quite good. There was…good feedback from the researchers. We had…good participation. So, we are in the process of increasing the competences within this field. But the main point, related to that competence, is local; okay, they could get back to us, but it takes some time and it costs something. So, these are the barriers. I think there are barriers related to the fact they don’t realize it is important or think [whether] it is important or not.” (P41, clinical trial support) “So I think the barrier is often [a] lack of experience, and I think if you are involved in several trials, like me, you get sort of better at also identifying that. Because I do think that the systematic review for planning a trial is not the same as what you would do in order to submit a manuscript…or to kind of summarize literature. It can be…very useful, all the data that you identify, but it is not quite the same exercise. So, I think the barriers in a way are the same as for getting into trials in general, in that, you know, it is not something you can sort of teach yourself…you have to work with the people who are experienced trialists, and you learn from them. And in my experience, that is…the only sensible way to learn about how to do trials and how to do them efficiently….So the main barrier, I think, is that people who plan trials may not have access to experts who understand how you need to look at the literature in order to identify the need for a trial or to inform your trial design.” (P44, primary investigator)
Lack of enforcement	
Clinical trial funders	“You know, I don’t like requirements, because I think this is something [that] is against the thinking we have at an academic institution; you know, having the freedom. But it could be a suggestion, you know. But actually, the problem [is], it’s not always feasible…it may be misleading if you make such a strong statement.” (P17, clinical trial funder) “I think generally, the [funder] has a little bit [of] the approach [that] we evaluate, but we don’t define what they have to provide us specifically for that evaluation criteria. So we do have…evaluation criteria of whether that is an unmet medical need and if it’s clinically relevant. But what they need to provide us to show this…we consider they know that better than us.” (P48, clinical trial funder)
Ethics committees	“Well, they look at that, we look at what kind of literature they looked at. We don’t do that for finding, like, scientific excellence. We just look: What did they do?...But we’re not that familiar with every field or every department…I mean, this is the responsibility of the researcher.” (P3, ethics committee) “This is also not our task, I think, to fully understand the specifics of a research topic. I think it must be in a way that we can follow the considerations of the researcher and to make a judgement whether this is useful or not. But not in every detail; this is impossible. We are not the experts. Usually, it is the researcher, and it must be clear that the question has some kind of relevance. But we cannot assess all the details. This is at the end [up] to the researcher.” (P27, ethics committee) “We don’t require [it], because then we could skip nearly all the investigations. So, I mean, this very often…with this, they call it weak studies; at the end, it is the problem of the investigator, then when they are going to publish. Then these are all the studies [that] will not be published in the high-rank journals, because [this is] then exactly the lack…they have in the beginning. As long as there [is] an understandable question they are asking and the methods they provide are able to answer this, okay. And if the scientific base or the evidence base of this is poor, yes, so what. As long as it is safe for the patients, or…this is [a lot] of retrospective studies where they are looking at data they have collected over the last 10 years, such [as] what studies.” (P45, ethics committee)
**Factors facilitating a systematic approach**	
Funders making systematic evidence synthesis mandatory	“That depends on the funders. Sure, if you’re talking about investigator-initiated trials, how does it make sense if you put up much public money without knowing what the current evidence is for the research question? That doesn’t make a lot of sense. I think you should request it, you know, whether you were requesting a formal meta-analysis, it depends on the subject. It might be difficult, or you come up with estimate[s] that are useless, because the endpoints are different, or the data [are] not there. But I mean, that you systematically collect the evidence before you start a new project, I think that should [be] done. Yes.” (P38, primary investigator) “They could do it because they [essentially give] the money, so they pay for it. So, we are not paying;…essentially, we are looking at safety for patients.” (P45, ethics committee)
Better support from academic institutions and associated clinical trial support organizations	“Well, I think of course, a funding body could make it a criterion…a must-have [criterion]. So, they can say, ‘Whenever you submit a clinical trial to us, please include a systematic review.’ And on the other hand, I think the academic institutions might then help investigators in doing such a review on a manageable level. So, I think they could be promoting that as well, because if you have this 2-[sided]-approach, you could convince funding to say, ‘Look, if there is an application, a clinical trial, it must include a systematic review.’ And on the other side, provide the assistance for the academic institutions to help their researchers to do that.” (P31, clinical trial funder)

In contrast, international funders reported a clear expectation that academic clinical trial proposals should include some form of systematic evidence synthesis (Table 2). However, participants reported large variations in terms of the type of evidence synthesis required and when it should be required.

**Table 2.** International Funders’ Views on Reviewing the Existing Research

Theme	Example quote (participant identifier, group)
**Current practice**	
Type of evidence synthesis required	
Formal systematic reviews required	“But it’s really around virtually late-stage research, comparative research relevant to the health care system. So, you know, it can’t be something that’s a great idea but has never been tried out in a clinical setting….If there isn’t a systematic review that directly addresses the problem, then we expect that to be put forward….I think the reason why we have this approach…[is that] there is a huge emphasis there on public funding, public accountability involving patients with open decision making. And so the systematic review is a part of opening up the scientific process to public scrutiny.” (P52, international funder) “I would say the same, and if someone was looking for 2 million pounds to run a definitive trial, then I would expect the full systematic review. But if somebody needs, you know, 80 000 to 100 000 [currency] for some feasibility work, you wouldn’t expect them to be doing work that costs as much as that to frame their application. But at the same time, you’d want them to demonstrate to the satisfaction of an expert panel that they are exploring a question that is not yet answered and which needs to be answered.” (P55, international funder)
Systematic assessments required	“So in terms of the evidence that we look for, we are asking an applicant to provide evidence that there is a need for the intervention and the background kind of literature search that supports that. So we don’t necessarily mandate that there needs to be a kind of formal systematic review or, you know, published review. What we are looking for is that there has been some systematic search of the literature and appraisal that supports the need for the intervention and for the way that the team is proposing to develop or test that intervention.” (P49, international funder) “No, we don’t ask them to perform a Cochrane review….We expect them to be a bit systematic in the sense that they need to provide [an] algorithm. They need to provide the match term, the keywords, you know, but it would not qualify as a systematic review in the sense that you would think about it when you would do a guideline, for instance, because that’s way too much work. So they need to focus on really, you know, the important trials, good quality, high quality, and if there is no evidence, then they need to give what they have found; but it’s not like the systematic review you would normally do, because that…takes months.” (P53, international funder)
Left to investigators	“It’s pretty much…I mean, there is guidance…we have peer review templates and then funding board committee member assessment templates, and there is guidance on those matters; we would tend to regard them as more expert than us, and so beyond [that], making it clear that that’s what we’re asking them to do…the researchers to establish that there is a research gap and then to bring any knowledge that they have to bear on that question. We leave the specifics to them.” (P55, international funder) “If you are working with templates, giving them too much detail, then sometimes it’s easy for them to check boxes—‘Oh, I have done that, done, done, done, finished.’ And you want to let them be creative and [have] their own thoughts about why should they do that. So that’s why we’re not getting too much detail in it. We just give the limited information….So we have no limitations on the maximum number of pages of the application…that’s why we leave it free.” (P56, international funder)
When evidence synthesis is required	
Preapplication stage	“We have a 2-step process: first they have to hand in a draft proposal, and if this get[s] recommended, they have to hand in a full proposal, and we always [ask them] to do literature research, so in the draft proposal…they have to lay down their literature research, they have to show their research strategy and the literature strategy.…The first step, the draft proposal, they really have to show that there is evidence that this clinical trial needs to be done and they will get an answer out of this clinical trial. This is the first step, and the second step, [if] it’s clear that there is a lack of knowledge,…then they’re going more into the detail into the clinical trial design….In the first step, it’s really…a question [of] is there really a gap of knowledge and do they have the right hypothesis, and is it possible to answer the question with the kind of trial they decided to use? That’s the first step.” (P57, international funder)
Full application stage	“Because in the first step, we get a lot of proposals. So it’s going to one-third, one-third, one-third. So in the common rounds for our program, we’ve got yearly 130 proposals, and, well, about 30 to 40 get a positive advise to work their application, to a full application; and from the [30 to 40] there is one-third who get the funding. So from 130 you go to 10, 15 funding proposals, and if you are asking for the literature review at the proposal, it takes much time from the applicants and also from the committee to get in detail for all of this information; but in the proposal, at the first step, they also can list kind of [on] which references this proposal is based.” (P56, international funder) “To be honest, I think the applicants kind of get it, as evidenced by the applications we [are] seeing now. In fairness, because it is introduced at full application stage, not at the preapplication, there may be a crop of people that just never make it through short listing that also would have struggled with this aspect….And it’s a good question; it’s not something that you just rattle off in a couple of weeks. I suppose what we are looking for in a preapplication…we need…to filter, you know, at that stage because [there] are simply too many applications otherwise. On the one hand, we need to filter out, but on the other hand, there is also always the balance of what information do you need to make a decision and how much work do you put on applicants if they are not going to be funded [in] the end?” (P51, international funder) “I think just reflecting on that, I completely agree in that, you know, if the evidence isn’t there to support the trial, it’s a no-go [criterion], but we haven’t put any funding into it. So I agree that it would be much more beneficial to have it up front [in the] initial application, but I think there is, I guess, some sympathy to the shortness of the application at the outline stage.” (P54, international funder)
**Barriers to a systematic approach**	
Practical challenges: time and resources	“So I think one of the barriers would be the time and resource[s] to do systematic reviews. So I suppose that’s partly why we don’t demand a formal systematic review. Because, you know, applicants spend a lot of time in preparing applications, and that’s quite a lot of work in itself. A proper systematic review, well, you’re probably talking 30, 40, 50 thousand [currency] to do that, which is unfunded. So I think that if it was an absolute requirement for a formal published systematic review, then I think that would create kind of problems. There might be a kind of a perverse sort of effect in that. We might prevent some kind of quite innovative research, too. It is very rigorous, but mandating that, I am not sure we necessarily have the desired effect.” (P49, international funder) “Well, I think one barrier is cost. Not all funders provide funding for systematic reviews.” (P55, international funder) “It’s the time that the applicants have to do the application and also the chance of getting funding in our program. Only one-third will end up with funding. So it’s also always a balance which effort you give before the application, and there is a chance of getting one also free to end up with…financial support for your study. So that’s also the balance we have….You won’t ask too much, but it should be enough information…that the committee [can] make a good decision; is this study needed at this moment?” (P56, international funder) “You know, in terms of barriers, I think we would expect that someone…applying for a large sum of money [would] have the…you know, if they are going to be able to run a large multicenter trial, we would expect them to have the resources to do that sort of preparatory work.” (P52, international funder)
**Factors facilitating a systematic approach**	
Established evidence synthesis community	“Well, so in [our country], I think we benefit from a very strong synthesis community, right? We have…these entities that part of their mandate was training people on these methodologies, right?...Because I think a lot of the people who want to do clinical trials, you know, or RCTs, are in the intervention in the clinical space, but systematic review is not necessarily something that’s part of their first training, right? And that comes in the mentoring. And so to me, there is a skill set to being able to do that; it’s a capacity that you need to be able to build and, you know, I talked about things like critical appraisal, understanding how to evaluate the evidence and include what and exclude what and why and, you know, properly shape your questions so you are able to clearly answer. So to me, it’s really a capacity thing, and one thing we’ve benefited from in [our country] is that we do have a strong community on that, and you do see the collaboration and, you know, we have a couple of methods group[s] that continue to evolve…the methods side, which is why…there [are] all kinds of other, you know, knowledge synthesis and mixed methods and all those things....I think it’s important not to underestimate that, because it’s an important part of understanding the literature and how it all fits together to be able to contextualize what you want to do.” (P50, international funder) “I mean, I work within an environment where there are many people with that expertise. I mean, most university hospitals…it’s not a problem….People know how to do systematic reviews....I think what would help improve the quality is more information. You know, I think just encouraging people to up their game in terms of methodology. So yeah, I mean, paying attention to what other people are doing, collaborating with other people, having good peer review of funding application, of publication, manuscripts for publication, that sort of thing.” (P52, international funder)
Guidance	
Providing more explicit requirements to investigators	“Yeah, I think funders. You can…[give] clear guidelines. You know, I think the more information you give them—you know, ‘Please check minimum this, this, and this database’—the more information you give, the easier it gets for them. So from funders…and I guess from, you know…publishers, journal editors, they also would like you to kind of perform at least…I mean, you need to compare your results based on literature...but from funders definitely.” (P53, international funder)
Improving knowledge of the different types of evidence synthesis	“I think it would be helpful, and this may well be out there, to go and have a look at some work on the whole kind of the suite of different kind of reviews that are available and information on reasonable standardized costs information on the benefits and the drawbacks of each. I think that would help people in my kind of role to improve our practice and our expectations of researchers and our assessors. But it is something we’ve become aware of recently as a knowledge gap and one that we ourselves need to fill if we’re going to do our job better.” (P55, international funder) “I think the research community needs to come together and provide evidence as to, you know, how useful different ways are. That’s really helpful. If there is a series of publications that suggest that actually rapid reviews pick up 99 percent of what’s there and, you know, spending more money on and time on systematic reviews is unnecessary, I think we will take that on board. But it’s always going to be context specific and risk specific.” (P52, international funder)

Abbreviation: RCT, randomized clinical trial.

#### Types of Evidence Assessment Required

The level of comprehensiveness that participants expected in summaries of existing evidence typically depended on the type of clinical trials being funded. A funder of large, late-stage multicenter clinical trials reported that they expected a formal systematic review to be provided; this was seen as important owing to the focus on public funding and accountability in their country, with systematic reviews being a part of opening the scientific process to public scrutiny. However, most funders of smaller, earlier-stage clinical trials reported that they did not mandate formal systematic reviews and only required a systematic search of the literature (including the search strategy and databases used). Nevertheless, 2 international funders who were members of the Ensuring Value in Research Funders’ Forum reported that they did not have explicit requirements regarding evidence assessment and left it to investigators what to provide.

#### When Evidence Assessment Is Required

Most funders reported having a 2-step funding process. Although 1 funder required systematic evidence assessments to be provided at the preapplication stage, most funders required it at the full application stage. Participants from the latter group reported that the key reason for requesting the evidence assessment at the second step was the limited space available in preapplications and the desire to reduce the work of the funder and the applicants who would not be funded.

### Barriers to an Evidence-Based Approach

Swiss participants identified 3 key barriers that may explain why systematic reviews are used infrequently.

- The first barrier was investigator perceptions. Participants reported that various views of principal investigators led them to not conduct systematic reviews before a new trial, including the view that they were experts in the field and already knew the relevant research; the view that their study was very innovative, and therefore, a systematic review did not make sense because of the limited evidence available; and the belief that an evidence-based approach could be achieved without a formal systematic review.
- The second barrier was practical challenges. Some practical challenges were also identified by participants as reasons why systematic reviews were not conducted, including systematic reviews being very time consuming; a lack of specific funds, which led to systematic reviews often being done without funding; insufficient personnel with the right expertise to conduct systematic reviews; and a lack of awareness and knowledge of systematic reviews among investigators and funding panels.
- The third barrier was lack of enforcement. Participants also reported that none of the funders of academic clinical trials in Switzerland were currently requiring a systematic review, that the method for reviewing the existing research was left to principal investigators, and that funders relied on the expertise of reviewers to evaluate whether the information presented in applications was sufficient. Participants also reported that ethics committees did not require systematic reviews and that although ethics committees checked whether the literature review conducted was generally sufficient and might ask for more information if the review was clearly inadequate, they ultimately saw this as the responsibility of the principal investigator and outside the task of ethics committees.

International funders did not report perceiving substantial barriers. Investigators were seen to have sufficient awareness and knowledge and generally accepted the funder’s expectations regarding evidence assessment. The practical challenges of time and resources were identified as the main barriers to formal systematic reviews. Because formal systematic reviews were perceived as time consuming and typically unfunded, these participants reported that mandating them could create problems and undermine innovative research. However, a funder of larger clinical trials noted that if an investigator was applying for a substantial sum of money to run a large multicenter clinical trial, they would expect the investigator to have the resources to do this sort of preparatory work.

### Factors Facilitating an Evidence-Based Approach

Many Swiss participants reported that the best way to facilitate systematic evidence assessments was for funders to make them mandatory. Although participants said they wanted such a requirement to be feasible and flexible, many reported that funders were the most suitable stakeholder to enforce evidence assessments and said that knowing what the current evidence is for a research question should be a prerequisite for academic clinical trials, particularly if large amounts of public funds are involved. However, many participants also acknowledged that any such requirement would need to be combined with better support from academic institutions and associated clinical trial support organizations.

In contrast, multiple international funders highlighted how evidence assessment communities had become firmly established in their countries, with sufficient capacity now existing for training, expertise, and collaboration, which had been important in promoting a systematic approach. Moving forward, some funders said that it would be helpful to provide investigators with more explicit requirements to clarify the level of comprehensiveness expected. However, funders identified a need to improve their own knowledge of the different types of evidence assessment (eg, formal systematic reviews vs rapid reviews) and for more research on the advantages and disadvantages of each approach.

## Discussion

To our knowledge, this is the first qualitative study to examine the barriers to and factors facilitating an evidence-based research approach with several stakeholders in the context of academic clinical trials. This study found general agreement among Swiss stakeholders and international funders that new clinical trials should be justified by a systematic evidence assessment, but there seemed to be substantial variation among stakeholders in terms of the expected comprehensiveness and transparency of evidence assessments. Formal systematic reviews are currently not expected and not conducted before most clinical trials, but most international funders expect a systematic search for the existing evidence. Whereas time and resources were reported by all participants as barriers to conducting systematic reviews, the Swiss research ecosystem was reported not to be as supportive of a systematic approach compared with international settings. Moving toward a more evidence-based research approach in academic clinical trials requires changes on multiple levels.

On an individual level, we identified a lack of awareness and knowledge of the importance of systematic reviews as well as a lack of skills to conduct systematic reviews. Providing training and integrating courses into the curricula of life sciences to build capacity in evidence-based research as well as more support from academic institutions (eg, information specialists in libraries where systematic literature searches are conducted) could help to overcome these barriers.^38,39^ In addition, it seems necessary to raise awareness within the scientific community that expert opinion and narrative reviews are not enough to implement evidence-based research. They have a high risk of being distorted by confirmation bias—the tendency to search and interpret information in a way that supports one’s own beliefs—and therefore are not sufficient to foster transparent evidence-based research.^40^

At an organizational level, resource constraints (eg, time and money) often hinder the conduct of comprehensive systematic reviews. Conducting a comprehensive systematic review can take up to 1 to 2 years.^41^ Rapid reviews, “a form of knowledge synthesis that accelerates the process of conducting a traditional systematic review through streamlining or omitting specific methods to produce evidence for stakeholders in a resource-efficient manner,”^42^ have emerged as pragmatic alternatives to systematic reviews and could enable primary researchers to sustainably adopt an evidence-based approach.^43,44^ Conducting a systematic review is (if at all) a prerequisite during the application stage, meaning that the update or conduct of a systematic review is often not funded. Thus, researchers may opt to apply for a clinical trial grant without putting unpaid resources into the production of a systematic review. Funders could consider financially supporting the production of systematic reviews or providing grant application funds. Although this might cost money initially, it could save money and resources by avoiding research waste in the long run.

On a political level, the lack of funders and ethics committees requiring systematic reviews was perceived by the study participants as a barrier. Making systematic evidence assessments mandatory for funding and ethics approval emerged as the most promising factor to facilitate implementation of evidence-based research. According to the National Institutes of Health Research in the UK, specifically requiring systematic reviews to justify the need for a new large multicenter trial can increase their use to nearly 100%.^17^ For smaller, earlier-stage clinical trials, funders could also explicitly clarify the requirements with respect to a systematic evidence assessment (eg, requesting at least a documented search strategy with searched databases). Ethics committees have also been called on to more vigorously advocate for an evidence-based approach.^45^

This study fills a research gap because, to our knowledge, other studies generally investigated the “natural history of conducting and reporting clinical trials” in interviews, with trialists only touching on “insufficient reference to previous research” when planning a new trial,^46^ or they specifically focused on a survey of methodologists on their opinions about a conditional trial design framework using network meta-analysis for the planning of new trials.^47^ The current study contributes to the findings of the preliminary survey of methodologists by Clayton et al^18^ by including perceptions of various stakeholders outside the UK and Ireland and by conducting in-depth interviews, which resulted in a more thorough understanding of barriers to and factors facilitating an evidence-based approach when designing a new trial.

### Limitations

This study has limitations. First, this qualitative study did not use a random sample of stakeholders. However, we included a range of experts who had direct experience with systematic evidence assessment in academic clinical trials, which makes it likely that this study captured key aspects of a multisided issue and provided applicable results. Second, a bias might have existed toward the reporting of socially desirable attitudes.^48^ Given that the results were critical of current practice, we believe such a bias is limited. Third, with the exception of funders, all participants were from Switzerland, compromising the generalizability of the findings to some degree. However, the findings are generally in line with existing research,^18^ and probing current practices of funders from different countries that have the reputation of endorsing an evidence-based approach and contrasting their views with Swiss stakeholders has yielded rich findings about knowledge gaps and insufficient clarity of funder requirements regarding evidence assessments. There appear to be a number of common issues across countries, which likely make our findings of interest to many countries.

## Conclusions

In this qualitative study, there was general agreement among Swiss stakeholders and international funders that new clinical trials should be justified by a systematic evidence assessment. However, investigators reported that barriers on individual, organizational, and political levels still regularly kept them from implementing these assessments. In their role as gatekeepers, funding agencies and ethics committees are in a position to enforce an evidence-based research approach by making it mandatory for new clinical trials.^17,45^ In addition, universities should train students and researchers in evidence-based methods and raise awareness of the importance of a systematic and transparent approach to justify new trials.

## Notes

Supplement. **eAppendix 1.** Systematic Search for Prior Qualitative Studies **eAppendix 2.** Extended Methods **eAppendix 3.** Interview Guides
