# Supplementary material for: Barriers and Facilitating Factors for Conducting Systematic Evidence Assessments in Academic Clinical Trials
Source: JAMA Netw Open. 2021 Nov 30;4(11):e2136577. doi: 10.1001/jamanetworkopen.2021.36577 (PMC8634056; doi:10.1001/jamanetworkopen.2021.36577)
Supplement: Supplement. — eAppendix 1. Systematic Search for Prior Qualitative Studies eAppendix 2. Extended Methods eAppendix 3. Interview Guides [file jamanetwopen-e2136577-s001.pdf]

Supplementary Online Content

McLennan S, Nussbaumer-Streit B, Hemkens LG, Briel M. Barriers and facilitating factors for conducting systematic evidence assessments in academic clinical trials. *JAMA Netw Open*. 2021;4(11):e2136577. doi:10.1001/jamanetworkopen.2021.36577

eAppendix 1. Systematic Search for Prior Qualitative Studies
eAppendix 2. Extended Methods
eAppendix 3. Interview Guides

This supplementary material has been provided by the authors to give readers additional information about their work.

© 2021 McLennan S et al. *JAMA Network Open*.

Supplemental Material

Supplemental Text

eAppendix 1: Systematic search for prior qualitative studies

We systematically searched all articles citing at least one of 6 key articles [1-6] on the use of systematic reviews in clinical trial research. These articles were identified in a recent report published by the Evidence-Based Research Network [7] and indexed in Web of Science. We automatically searched the titles and abstracts of these citing articles for the keywords “interview”, “qualitative”, “stakeholder”, “funder”, and “survey” and perused all identified articles. We identified one survey study aiming to examine barriers and facilitators of conducting systematic evidence assessments to justify and inform the design of new clinical trials [8].
Date of last search: 15 July 2021

References

[1] Clarke M, Alderson P, Chalmers I (2002) Discussion sections in reports of controlled trials published in general medical journals. JAMA 287: 2799-2801

[2] Clarke M, Hopewell S, Chalmers I (2007) Reports of clinical trials should begin and end with up-to-date systematic reviews of other relevant evidence: a status report. Journal of the Royal Society of Medicine 100: 187-190

[3] Clarke M, Hopewell S, Chalmers I (2010) Clinical trials should begin and end with systematic reviews of relevant evidence: 12 years and waiting. Lancet 376: 20-21

[4] Goudie AC, Sutton AJ, Jones DR, Donald A (2010) Empirical assessment suggests that existing evidence could be used more fully in designing randomized controlled trials. J Clin Epidemiol 63: 983-991

[5] Helfer B, Prosser A, Samara MT, et al. (2015) Recent meta-analyses neglect previous systematic reviews and meta-analyses about the same topic: a systematic examination. BMC Med 13: 82

[6] Jones AP, Conroy E, Williamson PR, Clarke M, Gamble C (2013) The use of systematic reviews in the planning, design and conduct of randomised trials: a retrospective cohort of NIHR HTA funded trials. BMC Med Res Methodol 13: 50

[7] Lund H, Juhl CB, Nørgaard B, et al. (2021) Evidence-Based Research Series-Paper 2 : Using an Evidence-Based Research approach before a new study is conducted to ensure value. Journal of Clinical Epidemiology 129: 158-166

[8] Clayton GL, Smith IL, Higgins JPT, et al. (2017) The INVEST project: investigating the use of evidence synthesis in the design and analysis of clinical trials. Trials 18: 219

© 2021 McLennan S et al. *JAMA Network Open*.

eAppendix 2: Extended Methods

The methods of the study are presented in accordance with the "Consolidated criteria for reporting qualitative research" (COREQ) [1].

Research team and reflexivity

Personal characteristics: Interviews were primarily conducted by S.M., a male senior researcher in biomedical ethics. One interview was conducted by M.B., a male physician and senior scientist in clinical epidemiology. Both interviewers have longstanding experience with qualitative research in the context of clinical research and evidence-based medicine [2-17].

Relationship with participants: The interviewers had already had contact with a number of the Swiss stakeholders from previous research studies. Otherwise, no relationship was established between the interviewers and the other participants prior to the study and participants received limited information about interviewers. There was no hierarchical relationship between the interviewers and the study participants.

Study design

Theoretical framework: The theoretical framework employed in this study was conventional content analysis [18].

Participant selection: Participants were primarily selected through purposive sampling [19]; in order to ensure sample diversity according to predetermined factors (e.g. field of expertise). Experts who were considered to be knowledgeable about the subject and capable of representing the views of his or her peers were identified through discussions within the research team and wider contacts.. Additional participants were identified using snowball sampling [20]. Participants were contacted by email and suitable dates for an interview were found with those willing to participate. The study consists of 57 participants from two different samples. In the first sample, forty-eight Swiss stakeholders from four different groups were recruited: primary investigators (PIs) of investigator-initiated clinical trials (n=27), funders and sponsors (n=9), clinical trial support organisations (n=6), and ethics committee members (n=6). In the second sample, nine international funders from North America and Europe with a reputation for implementing an evidence-based approach for clinical trials were recruited. Four people refused to participate because they did not think they were the suitable person or because of workload issues.

Setting: Interviews were conducted between February and August 2020 with Swiss stakeholders, and between January and March 2021 with international funders. One participant provided their response in writing via email, the remaining interviews were conducted via a telephone or video call. All interviews were conducted in English. Only the participant and the researcher were present during the interview. In the Swiss sample, 75% (36/48) of participants were male presenting, and 25% (12/48) were female presenting. In the international funder sample, 56% (5/9) of participants were female presenting, and 44% (4/9) were male presenting.

Data collection: A researcher-developed semi-structured interview guide was developed for each group to guide the discussion (**Supplemental Text 3**). Based on the first two interviews that did not show any problems, it was decided that no further piloting or adaptation of the interview guides was necessary. No repeat interviews were carried out. Interviews were audio recorded, no field notes were taken. Interviews lasted an average of 29 minutes (range 12 minutes – 62 minutes) with Swiss stakeholders, and 30 minutes (range 22 minutes – 44 minutes) with international funders. After 57 interviews the question about data saturation arose and discussed by the research team [21]. It was concluded that no new substantively new themes were being expressed by the participants. . Transcriptions of the interviews were returned to all participants with an invitation for them to review the transcription and send any corrections or clarifications; eight responses were received with minor corrections to syntax.

Analysis and findings

Using the interview transcriptions in their original language, S.M. performed conventional content analysis with the assistance of the qualitative software MAXQDA v11. Analysis commenced while interviews were ongoing and used an iterative approach where initial codes identified common across participants as well as those unique to individuals were identified using a process of open coding, and developed as the analysis progressed. B.N.S., L.H., and M.B. reviewed the initial analysis to clarify and refine codes, and conversations among the investigators continued until coding differences were resolved and consensus was achieved. Findings are presented as higher- and lower-level categories in a coding frame.

References

1. Tong A, Sainsbury P, Craig J. Consolidated criteria for reporting qualitative research (COREQ): a 32-item checklist for interviews and focus groups. Int J Qual Health C 2007; 19: 349-57.
2. McLennan S, Griessbach A, Briel M. Practices and attitudes of Swiss stakeholders regarding investigator-initiated clinical trials funding acquisition and cost management. JAMA Network Open. 2021;4(6):e2111847.

© 2021 McLennan S et al. *JAMA Network Open*.

3. Briel M, Elger B, von Elm E, Satalkar P. Insufficient recruitment and premature discontinuation of clinical trials in Switzerland: qualitative study with trialists and other stakeholders. Swiss Med Wkly 2017;147:w14556.
4. Gloy V, McLennan S, Rinderknecht M, Ley B, Meier B, Driessen S, Gervasoni P, Hirschel B, Benkert P, Gilles I, von Elm E, Briel M. Uncertainties about the need for ethics approval in Switzerland: a mixed-methods study. Swiss Med Wkly. 2020 Aug 12;150:w20318. doi: 10.4414/smw.2020.20318. PMID: 32799307.
5. Briel M, Speich B, von Elm E, Gloy V. Comparison of randomized controlled trials discontinued or revised for poor recruitment and completed trials with the same research question: a matched qualitative study. Trials. 2019 Dec 30;20(1):800. doi: 10.1186/s13063-019-3957-4. PMID: 31888725; PMCID: PMC6937940.
6. von Niederhäusern B, Schandelmaier S, Mi Bonde M, Brunner N, Hemkens LG, Rutquist M, Bhatnagar N, Guyatt GH, Pauli-Magnus C, Briel M. Towards the development of a comprehensive framework: Qualitative systematic survey of definitions of clinical research quality. PLoS One. 2017 Jul 17;12(7):e0180635. doi: 10.1371/journal.pone.0180635. PMID: 28715491; PMCID: PMC5513422.
7. Kasenda B, Liu J, Jiang Y, Gajewski B, Wu C, von Elm E, Schandelmaier S, Moffa G, Trelle S, Schmitt AM, Herbrand AK, Gloy V, Speich B, Hopewell S, Hemkens LG, Sluka C, McGill K, Meade M, Cook D, Lamontagne F, Tréluyer JM, Haidich AB, Ioannidis JPA, Treweek S, Briel M. Prediction of RECRUITment In randomized clinical Trials (RECRUIT-IT)-rationale and design for an international collaborative study. Trials. 2020 Aug 21;21(1):731. doi: 10.1186/s13063-020-04666-8. PMID: 32825846; PMCID: PMC7441612.
8. McLennan S. Rejected Online Feedback from a Swiss Physician Rating Website between 2008 to 2017: Analysis of 2352 Ratings. Journal of Medical Internet Research. 2020;22(8):e18374.
9. McLennan S. The Content and Nature of Narrative Comments on Swiss Physician Rating Websites: Analysis of 849 Comments. Journal of Medical Internet Research. 2019;21(9):e14336.
10. McLennan S. The ethical oversight of learning health care activities in Switzerland: a qualitative study. International Journal for Quality in Health Care. 2019; 31;31(8):G81-G86.
11. McLennan S, Moore, J. New Zealand District Health Boards' Open Disclosure Policies: A Qualitative Review. Journal of Bioethical Inquiry. 2019; 16(1): 35-44.
12. McLennan S, Strech D, Kahrass H. Why are so few patients rating their physicians on German physician rating websites? A qualitative study. BMC Health Services Research. 2018; 18:670.
13. McLennan S, Kahrass H, Wieschowski S, Strech D, Langhof H. The spectrum of ethical issues in a Learning Health Care System: a systematic qualitative review. International Journal for Quality in Health Care. 2018; 30(3): 161–168.
14. McLennan S, Schwappach D, Harder Y, Staender S, Elger B. Patient Safety Issues in Office-based Surgery and Anaesthesia in Switzerland: A Qualitative Study. Zeitschrift für Evidenz, Fortbildung und Qualität im Gesundheitswesen. 2017; 125: 23-29.
15. Pless A*, McLennan S*, Nicca D, Shaw D, Elger B. Reasons why Nurses Decline Influenza Vaccination: a Qualitative Study. BMC Nursing. 2017:16: 20.
16. Pless A, Shaw D, McLennan S*, Elger E.* Nurses' Attitudes towards Enforced Measures to Increase Influenza Vaccination: a Qualitative Study. Influenza and Other Respiratory Viruses. 2017;11(3):247-253.
17. McLennan S, Diebold M, Rich LE, Elger B. Nurses' Perspectives Regarding the Disclosure of Errors to Patients: A Qualitative Study. International Journal of Nursing Studies. 2016;54:16-22.
18. Hsieh HF, Shannon SE. Three approaches to qualitative content analysis. Qualitative Health Res. 2005;15(9):1277–88.
19. Palinkas LA, Horwitz SM, Green CA, Wisdom JP, Duan N, Hoagwood K. Purposeful Sampling for Qualitative Data Collection and Analysis in Mixed Method Implementation Research. Adm Policy Ment Health. 2015; 42(5): 533-44.
20. Marshall MN. Sampling for qualitative research. Fam Pract. 1996;13(6):522-5.
21. Fusch PI, Ness LR. Are We There Yet? Data Saturation in Qualitative Research. The Qualitative Report. 2015; 20(9): 1408-1416.

© 2021 McLennan S et al. *JAMA Network Open*.

eAppendix 3: Interview guides

Primary investigators interview guide

1. To start, could you tell me a bit about the types and how many clinical trials you have conducted?
    a. Were these single or multi-center clinical trials?
2. When you are justifying and designing a new clinical trial, how do you collect information about existing research?
    a. If a systematic review is used:
        i. What were your reasons for using this approach?
        ii. Do you think a systematic review should always be used before a new clinical trial?
    b. If a systematic review is not used:
        i. Would you consider this to be an "evidence-based approach"?
        ii. What were your reasons for not using a systematic review?
        iii. Do you have any concerns about systematic reviews?
3. What do you think are the barriers to systematically reviewing prior evidence when planning a new clinical trial?
    a. What could facilitate a systematic approach?
    b. In your view, which stakeholders would be best suited to promote and endorse an evidence-based approach?

…

Funding agencies interview guide

1. To start, could you tell me a bit about the types and how many investigator-initiated clinical trials applications you receive each year?
    a. How many of these are single or multi-center clinical trials?
2. What would characterize for you an "evidence-based approach" when justifying and designing a new clinical trial?
    a. Do you think a systematic review should always be used before a new clinical trial? Does your institution enforce this? If not, why not?
    b. Does your institution support the use of systematic reviews?
        i. If yes, in what way do you endorse such an approach practically?
        ii. If no, why not?
    c. In your estimation, what proportion of clinical trial investigators use systematic reviews when justifying and designing a new trial?
3. What do you think are the barriers to systematically reviewing prior evidence when planning a new clinical trial?
    a. What could facilitate a systematic approach?
    b. In your view, which stakeholders would be best suited to promote and endorse an evidence-based approach?

…

Trial support organizations interview guide

1. To start, could you tell me a bit about the types and how many investigator-initiated clinical trials your organization supports each year?
    a. How many of these are single or multi-center clinical trials?
2. What would characterize for you an "evidence-based approach" when justifying and designing a new clinical trial?
    a. Do you think a systematic review should always be used before a new clinical trial?
    b. Does your institution support the use of systematic reviews?
        i. If yes, in what way do you endorse such an approach practically?
        ii. If no, why not?
    c. In your estimation, what proportion of clinical trial investigators use systematic reviews when justifying and designing a new trial?
3. What do you think are the barriers to systematically reviewing prior evidence when planning a new clinical trial?
    a. What could facilitate a systematic approach?
    b. In your view, which stakeholders would be best suited to promote and endorse an evidence-based approach?

…

© 2021 McLennan S et al. *JAMA Network Open*.

Ethics committees interview guide

1. To start, could you tell me a bit about the types and how many investigator-initiated clinical trials applications you receive each year?
    a. How many of these are single or multi-center clinical trials?
2. What would characterize for you an “evidence-based approach” when justifying and designing a new clinical trial?
    a. Do you think a systematic review should always be used before a new clinical trial? Does your institution enforce this? If not, why not?
    b. In your estimation, what proportion of clinical trial investigators use systematic reviews when justifying and designing a new trial?
3. What do you think are the barriers to systematically reviewing prior evidence when planning a new clinical trial?
    a. What could facilitate a systematic approach?
    b. In your view, which stakeholders would be best suited to promote and endorse an evidence-based approach?

…

International funders interview guide

1. What would characterize for you an “evidence-based approach” when justifying and designing a new clinical trial?
    a. Do you think a systematic review should always be used before a new clinical trial? If yes, why? If not, why not?
2. For how many years has your funding agency implemented the requirement of a systematic review to be referenced in the funding application for a new clinical trial?
    a. Were there any positive or negative reactions from researchers or other stakeholder groups such as ethics committees, clinical trial units, other academic institutions or even industry upon this new requirement?
3. What do you think are the barriers to systematically reviewing prior evidence when planning a new clinical trial for the various stakeholders involved in clinical trials? (PIs, funders, others)
    a. What could facilitate a systematic approach?
    b. In your view, which stakeholders would be best suited to promote and endorse an evidence-based approach?
4. What do you know about “rapid reviews”? Do you think this could be a useful means to facilitate an evidence-based approach for clinical trials?
5. What do you see as important steps to sustainably implement an evidence-based approach for clinical trials?
6. Do you know of any ethics committees requiring a reference to a systematic review on the existing evidence for the approval of a new clinical trial protocol?

© 2021 McLennan S et al. *JAMA Network Open*.
